# Strengthening the TB response with artificial intelligence and the right to health

**DOI:** 10.5588/ijtldopen.25.0271

**Published:** 2025-09-10

**Authors:** B. Citro, S. Zaidi, J. Malar, O. Klymenko, B. Hermawan

**Affiliations:** ^1^ Independent researcher, Chicago, USA;; ^2^ Stop TB Partnership, Geneva, Switzerland;; ^3^ TBpeople Ukraine, Kyiv, Ukraine;; ^4^ Perkumpulan Organisasi Pasien Tuberkulosis Indonesia, Jakarta, Indonesia.

**Keywords:** tuberculosis, AI, computer-aided detection, community-led monitoring, chatbots, GPT-4

Dear Editor,

Artificial intelligence (AI) is playing an increasingly pivotal role in the global TB response. AI presents opportunities to enhance prevention, screening, diagnosis and treatment while augmenting disease hotspot mapping, TB literacy, and community engagement. In some cases, AI-based technologies are already accelerating efforts to end TB in hard-to-reach populations and democratizing access to care and information. Yet the same technologies pose risks to the rights of people affected by TB. These risks stem from concerns about data privacy, algorithmic bias, safety and efficacy. The substantial costs associated with operating and maintaining emerging AI technologies for health and their reliance on robust digital infrastructure and internet connectivity also raise concerns about their accessibility in high-burden countries. It is imperative that we protect the right to health of people with TB while leveraging new technologies to strengthen the disease response.

The right to health is central to the global TB response. It is embedded in the WHO End TB Strategy, the Global Fund’s 2023–2028 Strategy, and the Stop TB Partnership’s Global Plan to End TB, 2023–2030, and it forms core commitments in the 2023 Political Declaration of the United Nations High-Level Meeting on TB. In 2021, we published a review of 20 country-level assessments of legal, policy, and socioeconomic issues that inhibit access to quality TB services.^1^ The review employed seven key dimensions of the right to health under international law with a particular focus on the availability, accessibility, acceptability, and quality of TB services (AAAQ). These dimensions provide a framework to consider how AI-based technologies impact the right to health of people with TB.

The Table contains a snapshot of AI-based technologies in the TB response. Perhaps the most established use of AI in the TB response is computer-aided detection (CAD) for chest X-ray screening. Deep artificial neural networks are applied in CAD tools to analyze X-rays for signs of TB, circumventing traditional problems with image interpretation, including a shortage of radiologists and inter- and intra-reader variability.^2^ In 2021, the WHO conditionally recommended that CAD software ‘may be used in place of human readers for interpreting digital chest X-rays for screening and triage for TB disease’ in adolescents and adults.^3^ Many countries have since integrated CAD solutions into their TB screening programs, and the products have generally performed similarly or better than radiologists.^2^ In these respects, CAD tools may promote the right to health by increasing the availability and accessibility of TB screening services, particularly when combined with ultra-portable X-rays that extend access to vulnerable groups.^4^ However, like human radiologists, studies have shown CAD devices are less effective in certain subpopulations, including people living with HIV and older adults.^2,5^ As new products and versions come to market, there is also a limited understanding of how the underlying algorithms affect performance. Fehr et al. demonstrated that an update to a popular CAD tool, ©Delft Imaging’s CAD4TB, would have missed more people with TB when using the same threshold as previous versions during community-based screening in rural South Africa.^6^ Comparing three successive software versions, using the same triaging threshold, they found that the percentage of microbiologically confirmed TB cases missed would have been 20.2%, 11.1%, and 33.3%, respectively, for each update.^6^ Independent validation of CAD’s performance for other lung conditions is also limited. Most abnormal X-rays are not TB-related, and relying solely on CAD may hinder the detection of other illnesses if individuals are not assessed holistically. Additional data is needed to evaluate CAD’s performance for non-TB abnormalities.^7^

**Table.** Snapshot of artificial intelligence-based technologies in the TB response with their status and right to health impact.

Function/Use	AI technology	Status	Right to health impact
Screening and triage	Computer-aided detection (CAD) for chest X-ray	In programmatic use	Opportunity: May increase the availability and accessibility of high-quality TB screening services, including for vulnerable or marginalized groups.^2,4^ Risk: Studies raise concerns about the quality and acceptability of CAD tools, including for vulnerable groups and people suffering from other lung conditions, and their accessibility may be limited where internet connectivity is unreliable.^2,5–9^
Disease hotspot mapping	Machine learning-powered disease hotspot mapping software	In pilot use	Opportunity: May increase services accessibility, including for vulnerable groups.^10,11^ Risk: Collecting, storing, and processing sensitive datasets creates risks to privacy and confidentiality and may lead to discrimination if authorities or others misuse the data.
Treatment support and monitoring	Computer vision- and machine learning-enabled VDOT	In pilot use	Opportunity: May promote treatment acceptability and protect privacy and confidentiality by reducing reliance on facility-based DOT.^12^ Risk: May perpetuate DOT, which is linked to stigma, discrimination, accessibility barriers, and privacy breaches.^13^
TB literacy and services connectivity	Generative AI chatbots powered by large language models	In pilot use	Opportunity: May expand access to TB information and services.^14,15^ Risk: Raises privacy concerns related to the personal information users share with chatbots, quality and acceptability concerns due to bias and disparities in the information chatbots share, and accessibility and acceptability concerns for populations with limited digital literacy or internet connectivity.^16,17^
Community engagement	AI-enabled community-led monitoring platforms	In pilot use	Opportunity: May expand access to TB services and information, reduce TB-related stigma, increase community participation in TB decision-making, and extend access to legal remedies and accountability.^18^ Risk: Poses privacy, confidentiality, and non-discrimination risks if users’ data are breached, and accessibility depends on sustainable resources in high-burden contexts.

The issues around CAD tools implicate the quality and acceptability dimensions of the right to health, especially for vulnerable groups and people suffering from other lung conditions. By comparison, recent studies of widely used AI systems in U.S. healthcare showed that inherent bias in an algorithm used to allocate additional patient care likely contributed to worse health outcomes for minority patients,^8^ and an inpatient prediction model missed almost 70% of patients with a potentially deadly condition.^9^ Algorithmic bias and diminished efficacy in subpopulations may result from using non-diverse, unrepresentative datasets to train machine learning models. The internet connectivity required to operate CAD tools also raises concerns about their accessibility in low- and middle-income countries, particularly for rural or remote populations where connectivity is unstable.

Researchers and public health programs have used disease hotspot mapping software powered by machine-learning algorithms to predict areas with high TB prevalence in countries including Nigeria, Pakistan and the Philippines.^10^ Researchers in Nigeria trained a predictive Bayesian inference model on data generated during active case finding in four Nigerian states, along with a range of socioeconomic indicators, including population density, poverty level, HIV prevalence, child mortality rates, sanitation levels and nighttime lights.^11^ The retrospective study found that the yield in population clusters predicted by the machine learning model had TB positivity rates at least 1.75 times greater than in locations identified using standard methods. Hotspot mapping algorithms that help TB programs identify people with or at risk of TB may advance the right to health by promoting services accessibility, including for vulnerable groups. However, collecting, storing, and processing sensitive datasets risks impairing health-related freedoms, including privacy and confidentiality, and may contribute to stigma and discrimination if data is insufficiently protected or misused.

AI is also being piloted to support TB treatment adherence. Building on video directly observed therapy (VDOT; also referred to as ‘video-assisted therapy’), AI-based software utilizing computer vision and machine learning can confirm whether a person with TB is taking the appropriate drugs correctly.^12^ In one pilot, an AI-based video monitoring app sent nurses an automated SMS or email to follow up with their patients if they encountered challenges using VDOT.^12^ AI-based technologies that reduce reliance on facility-based DOT may promote treatment acceptability and protect health-related freedoms, including privacy and confidentiality. However, AI-based technologies perpetuating DOT’s use raise concerns because DOT is linked to stigma, discrimination, accessibility barriers and privacy breaches.^13^ Obtaining informed consent for AI-based adherence technologies is critical to ensuring users understand the associated risks. In doing so, healthcare providers must consider users’ digital literacy, particularly when explaining data privacy risks to members of vulnerable groups.

Generative AI chatbots are also increasingly being used for TB interventions. These large language models (LLMs) offer various functionalities, including sharing information about TB and supporting treatment adherence. The KNCV Tuberculosis Foundation piloted an LLM chatbot powered by OpenAI’s GPT-4 model to improve TB knowledge among the public, focusing on migrants with diverse language backgrounds in the Netherlands.^14^ Another recent study used GPT-4 to develop a ‘TB counselling assistant’.^15^ Using a modified Delphi consensus method, the study found that the chatbot provided accurate answers about TB epidemiology, clinical presentation, prevention, diagnosis and treatment. However, some of the information it shared was outdated or lacked context.^15^ While AI chatbots may expand access to TB information and services, they raise privacy concerns related to the personal information individuals share when using them. For example, researchers recently exposed a publicly available database that leaked sensitive user information, including chat histories, from DeepSeek, a leading generative AI chatbot used by millions of people.^16^ Bias and disparities in the content and accuracy of the information AI chatbots share raise further concerns about their quality and acceptability.^17^ Biased or inaccurate information from chatbots may result from using biased or unrepresentative datasets to train the LLMs. Additionally, the digital devices, internet connectivity, and digital literacy required to sustain the use of chatbots raise concerns about their accessibility and acceptability in high-burden countries, underscoring the global digital divide.

AI also powers community-led monitoring (CLM) to ensure a continuous supply of TB services in countries such as Indonesia and Ukraine. Under the Challenge Facility for Civil Society grant mechanism, Perkumpulan Organisasi Pasien Tuberkulosis Indonesia and TBpeople Ukraine utilize the OneImpact CLM platform, powered by an open-source LLM, to connect individuals with TB services, share critical information, and provide social, legal and psychological support, including peer-to-peer support.^18^ TBpeople Ukraine also plans to introduce an AI-powered virtual assistant and confidential video-supported therapy, among other features. AI-based CLM may advance the right to health by expanding access to TB services and information, reducing TB-related stigma, increasing community participation in TB decision-making, and extending access to legal remedies and accountability.^18^ Nonetheless, AI-powered CLM poses risks to privacy, confidentiality, and non-discrimination should users’ data be breached. Both organizations have also voiced concerns about the projects’ sustainability, despite the modest resources required to operate the platforms, raising concerns about their ongoing accessibility.

As AI is poised to transform the TB response, new tools are available to accelerate efforts to end TB by 2030. We must harness these new technologies to ensure access to high-quality services while protecting the right to health of people affected by TB. This includes training AI models (such as chatbots and CAD software) on datasets that accurately represent global TB demographics, and conducting longitudinal studies in high-burden settings to assess their sustained efficacy for those who need them most. We must also empower communities affected by TB to participate in developing rights-based standards to regulate these powerful technologies.
